# Pretreatment Diffusion-Weighted MRI Can Predict the Response to Neoadjuvant Chemotherapy in Patients with Nasopharyngeal Carcinoma

**DOI:** 10.1155/2015/307943

**Published:** 2015-08-27

**Authors:** Guo-Yi Zhang, Yue-Jian Wang, Jian-Ping Liu, Xin-Han Zhou, Zhi-Feng Xu, Xiang-Ping Chen, Tao Xu, Wei-Hong Wei, Yang Zhang, Ying Huang

**Affiliations:** ^1^Cancer Center, Cancer Research Institute, Foshan Hospital, Sun Yat-sen University, Foshan, Guangdong 528000, China; ^2^Imaging Diagnosis Center, Foshan Hospital, Sun Yat-sen University, Foshan, Guangdong 528000, China; ^3^Department of Radiation Oncology, State Key Laboratory of Oncology in Southern China, Collaborative Innovation Center of Cancer Medicine, Cancer Center, Sun Yat-sen University, Guangzhou, Guangdong 510060, China

## Abstract

*Purpose*. To explore the potential of diffusion-weighted (DW) magnetic resonance imaging (MRI) using apparent diffusion coefficient (ADC) for predicting the response to neoadjuvant chemotherapy in nasopharyngeal carcinoma (NPC). *Methods and Materials*. Ninety-two consecutive patients with NPC who underwent three cycles of neoadjuvant chemotherapy were retrospectively analyzed. DW and anatomical MRI were performed before and after neoadjuvant chemotherapy prior to radiotherapy. Pretreatment ADCs and percentage increases in ADC after chemotherapy were calculated for the primary lesions and metastatic adenopathies. Receiver operating characteristic curve analysis was used to select optimal pretreatment ADCs. *Results*. Pretreatment mean ADCs were significantly lower for responders than for nonresponders (primary lesions, *P* = 0.012; metastatic adenopathies, *P* = 0.013). Mean percentage increases in ADC were higher for responders than for nonresponders (primary lesions, *P* = 0.008; metastatic adenopathies, *P* < 0.001). The optimal pretreatment primary lesion and metastatic adenopathy ADCs for differentiating responders from nonresponders were 0.897 × 10^−3^ mm^2^/sec and 1.031 × 10^−3^ mm^2^/sec, respectively. *Conclusions*. NPC patients with low pretreatment ADCs tend to respond better to neoadjuvant chemotherapy. Pretreatment ADCs could be used as a new pretreatment imaging biomarker of response to neoadjuvant chemotherapy.

## 1. Introduction

Nasopharyngeal carcinoma (NPC) is one of the most common malignancies in Southeast Asia, especially in the southern provinces of China [1]. In patients with advanced NPC, neoadjuvant chemotherapy has been established as an alternative treatment for reducing tumor size, thereby facilitating local control and improving the disease-free survival rate, but not the overall survival rate, after administration of subsequent concurrent chemoradiotherapy [2–7]. However, not all patients respond to neoadjuvant chemotherapy, so identification of nonresponders at the time of pretreatment staging would allow the treatment regimens of individual patients to be modified or altered to concurrent chemoradiotherapy.

Currently, anatomical magnetic resonance imaging (MRI) is normally used to assess the tumor response after neoadjuvant chemotherapy in NPC [8, 9], though changes in the morphologically based measures of tumor diameter and volume occur relatively late during the treatment course. Furthermore, anatomical MRI cannot be used to predict the tumor response to neoadjuvant chemotherapy prior to treatment. The value of ^18^F-fluorodeoxyglucose positron emission tomography (FDG-PET) with respect to predicting tumor response is often hampered by its false-positive and expensive check cost [10, 11]. Therefore, it would be advantageous to identify novel imaging biomarkers that could predict the response to neoadjuvant chemotherapy prior to treatment in patients with NPC.

Diffusion-weighted (DW) MRI measures the diffusivity of water molecules within tissue extracellular spaces, which is quantified using apparent diffusion coefficients (ADCs). In general, intratumoral cell death induced by treatment increases water diffusion and leads to an increase in the ADC. Some early studies showed that DW MRI is helpful for predicting or detecting the response to chemoradiotherapy or radiosensitivity in head and neck carcinoma [12–19]. However, the efficacy of pretreatment ADCs obtained by DW MRI for predicting the response to neoadjuvant chemotherapy in NPC, or other head and neck carcinomas, has not been reported. The purpose of this study was to evaluate the potential of DW MRI using ADCs for predicting the response to neoadjuvant chemotherapy in patients with NPC.

## 2. Materials and Methods

### 2.1. Patients

This retrospective study was approved by the institutional review board and written informed consent was obtained from all participating patients or their next of kin. This study comprised 98 ethnic Chinese patients who were newly diagnosed with untreated and nonmetastatic NPC and who underwent both DW MRI and anatomical MRI before (baseline MRI) and after neoadjuvant chemotherapy but before radiotherapy (follow-up MRI) between March 2010 and April 2014. Median time between baseline MRI and the start of chemotherapy was 8 days (range, 2–21 days). Median time between the completion of chemotherapy and follow-up MRI was 16 days (range, 6–21 days). All 98 patients underwent the same neoadjuvant chemotherapeutic regimen and subsequently received intensity-modulated radiotherapy (IMRT). Six patients were later excluded from the study for the following reasons: four failed to complete three cycles of neoadjuvant chemotherapy, and two had inadequate DW MRI image quality due to excessive susceptibility or motion artifacts. The remaining 92 patients included 70 males (mean age, 46.5 years; range, 21–73 years) and 22 females (mean age, 44.3 years; range, 20–71 years). The World Health Organization (WHO) histologic type distribution was as follows: type I (*n* = 1), type II (*n* = 9), and type III (*n* = 82). According to the 2010 American Joint Committee on Cancer (AJCC) tumor-node-metastases (TNM) staging system [20], the prechemotherapy clinical stage distribution was as follows: stage III, 52 patients; IVA, 29; and IVB, 11.

Neoadjuvant chemotherapy consisted of docetaxel (60 mg/m^2^ or 65 mg/m^2^ d1), cisplatin (60 mg/m^2^ or 65 mg/m^2^ d1), and fluorouracil (600 mg/m^2^ or 650 mg/m^2^ d1–5) via intravenous infusion, repeated every 21 days for 3 cycles. After neoadjuvant chemotherapy, all patients were treated with definitive IMRT followed by concomitant chemotherapy (30–40 mg/m^2^ cisplatin or nedaplatin weekly).

### 2.2. Imaging Protocol

All patients underwent MRI using a 1.5 Tesla system (Signa CV/i, GE Healthcare, Milwaukee, WI, USA). The region from the suprasellar cistern to the inferior margin of the sternal end of the clavicle was examined using a head and neck combined coil. A T2-weighted fast spin-echo (FSE) sequence in the axial plane with a matrix of 512 × 512 and repetition time (TR)/echo time (TE) = 2889 ms/70.8 ms, a T1-weighted FSE sequence in the axial, coronal, and sagittal planes with a matrix of 560 × 560 and TR/TE = 627 ms/8.6 ms, and an echo-planar DWI sequence with a matrix of 224 × 224, TR/TE = 1360 ms/89.8 ms, and *b*-values of 0, 100, 500, and 1,000 s/mm^2^ were obtained before injection of contrast material. After intravenous injection of Gd-DTPA at a dose of 0.1 mmol/kg body weight, T1-weighted axial and sagittal sequences and T1-weighted fat-suppressed coronal sequences were performed sequentially, with parameters similar to those applied before the Gd-DTPA injection. The section thickness and interslice gap were 5 mm and 0 mm, respectively.

### 2.3. Image Assessment

The MRI scans were evaluated independently on a work station (Medi-PACS, Vancouver, Canada) by two radiologists, each with over 10 years of experience interpreting NPC MR images; any differences were resolved by consensus. Both radiologists were blinded to the therapeutic responses to neoadjuvant chemotherapy and other clinical findings.

Regions of interest (ROIs) were placed on the primary lesions and metastatic adenopathies on the images acquired using a *b*-value of 0 s/mm^2^ (excluding any necrotic regions identified with the aid of the T2-weighted and T1-weighted postcontrast MR images), and then the ROIs were automatically copied to the other *b*-value images by the software. Subsequently, all ROIs were merged per lesion for each *b*-value, and the average SI was calculated for the entire lesion. ADCs were derived using the following equation: ADC = −ln⁡[SI(*b*)/SI(0)]/*b*, where *b* is the diffusion weighting factor and SI(*b*) and SI(0) are the signal intensities with and without diffusion-sensitizing gradients, respectively [19]. Percentage increases in ADC (ADC%) were calculated as follows: ADC% = (ADCpost − ADCpre)/ADCpre × 100, where ADCpre and ADCpost are the pre- and posttreatment ADCs, respectively.

For each lesion, contours were drawn around the lesion border at each slice position based on anatomical MRI. Subsequently, the volume of each lesion was calculated using the following equation: (Σsurface at each slice position) ∗ (slice thickness + interslice gap). Percentage increases in volume (*V*%) were calculated as follows: *V*% = (*V*post − *V*pre)/*V*pre × 100, where *V*pre and *V*post are the pre- and posttreatment tumor volume, respectively.

The Response Evaluation Criteria in Solid Tumors (RECIST) guidelines were used to classify patients as responders or nonresponders on the basis of anatomical MRI [21]. A patient was considered to be a responder if all assessable lesions (both primary lesion and metastatic adenopathies) completely disappeared or partially reduced (≥30% in the sum of the maximal diameters) on the follow-up MRI. A patient was considered to be a nonresponder if measurable lesions were stable (<30% reduction or <20% increase in the sum of the maximal diameters) or progressed (≥20% increase in the area(s) of the original lesion(s) or the appearance of new lesions) on the follow-up MRI.

### 2.4. Statistical Analysis

Interreader agreement was evaluated with Cohen* K* coefficient for the image assessment. A* K* value of 0.4–0.6 indicated moderate agreement; 0.6–0.8, good agreement; and above 0.8, very good agreement [22]. The independent-samples* t*-test was used to compare the responders and nonresponders with respect to tumor volume (mean tumor volume of primary lesions and metastatic adenopathies before and after chemotherapy), pre- and posttreatment mean ADCs, and percentage increases in ADCs after chemotherapy. Fisher's exact test was used to compare age, sex, tumor patholog**y,** and clinical stage b**e**tween responders and nonresponders. Spearman's rank correlation was performed to evaluate the correlation between (a) the changes in ADCs and change in tumor volume on follow-up MRI and (b) pretreatment tumor ADCs and percentage change in tumor volume after chemotherapy.

To determine the optimal pretreatment ADC cutoff values with which to differentiate responders from nonresponders, receiver operating characteristic (ROC) curves and the areas under the curve (AUCs) were to evaluate the effectiveness of different criteria. The optimal cutoff value was defined as the value corresponding to the highest average sensitivity and specificity. The overall accuracy was represented by the AUC: the larger the area, the better the test. Sensitivity, specificity, and accuracy were calculated using the standard definitions [23]. SPSS version 16.0 (IBM, Armonk, NY, USA) was used for all data analyses, except for Fisher's exact test and ROC curve analysis that were performed using MedCalc software version 10.3.0.0 (MedCalc software, Mariakerke, Belgium).* P*-values < 0.05 were considered significant.

## 3. Results

### 3.1. Interobserver Agreement

In the per-lesion analysis, there was excellent agreement for the image assessment between observers 1 and 2, with* K* coefficients of 0.930 and 0.932 for pre- and posttreatment volume of primary lesions, 0.937 and 0.934 for pre- and posttreatment volume of metastatic adenopathies, 0.927 and 0.924 for pre- and posttreatment ADCs of primary lesions, and 0.931 and 0.928 for pre- and posttreatment ADCs of metastatic adenopathies. Any differences between observers 1 and 2 were resolved by consensus.

### 3.2. Treatment Outcomes

After completion of neoadjuvant chemotherapy, the primary tumor treatment responses were distributed as follows: complete resolution, 24 (26.1%) patients; partial resolution, 55 (59.8%) patients; and stability, 13 (14.1%) patients. The treatment responses of the metastatic cervical lymph nodes were distributed as follows: complete resolution, 44 (55.0%) patients; partial resolution, 26 (32.5%) patients; and stability, 10 (12.5%) patients. When the treatment responses of the primary tumor and metastatic cervical lymph nodes were considered together, 76 (82.6%) of the 92 patients were categorized as responders, and 16 (17.4%) were categorized as nonresponders (Table 1). No significant differences were observed between responders and nonresponders with respect to age, sex, tumor histology, or clinical stage (Table 2).

### 3.3. DW MRI and Tumor Volume

The mean tumor volumes of the primary lesions and metastatic adenopathies at prechemotherapy MRI were 37.2 cm^3^ (median, 36.6 cm^3^; range, 3.8–96.5 cm^3^) and 18.4 cm^3^ (median, 16.2 cm^3^; range, 1.3–49 cm^3^), respectively. No significant difference was observed between responders and nonresponders in terms of the mean pretreatment tumor volume (primary lesions, 36.6 cm^3^ ± 2.7 (standard error) versus 40.4 cm^3^ ± 6.7,* P* = 0.770; metastatic adenopathies, 18.6 cm^3^ ± 1.7 versus 19.5 cm^3^ ± 4.5,* P* = 0.906). However, after completion of neoadjuvant chemotherapy, responders had a smaller mean tumor volume than nonresponders (primary lesions, 9.2 cm^3^ ± 1.2 versus 28.0 cm^3^ ± 4.7,* P* = 0.014; metastatic adenopathies, 2.6 cm^3^ ± 0.5 versus 12.6 cm^3^ ± 2.9,* P* = 0.003) (Table 3).

**Figure 1:**
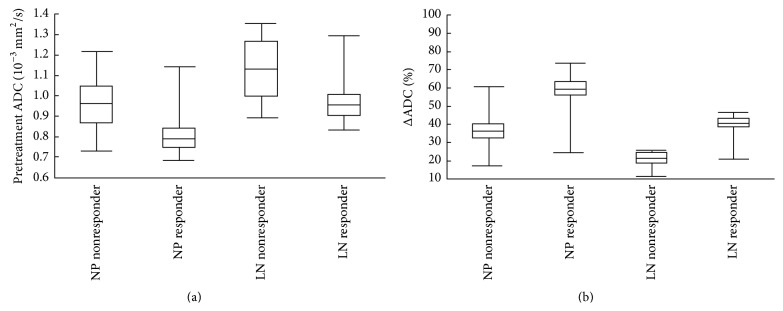
(a) Comparison of pretreatment ADCs for responders and nonresponders in patients with NPC. Responders had significantly lower pretreatment ADCs (primary lesions,* P* = 0.012; metastatic adenopathies,* P* = 0.013). (b) Comparison of ΔADCs for responders and nonresponders. Responders had significantly higher ΔADCs (primary lesions,* P* = 0.008; metastatic adenopathies,* P* < 0.001). Box-whisker plots are presented with median (-), interquartile range (box), and minima/maxima (-). NP = nasopharynx; LN = regional neck lymph nodes.

**Figure 2:**
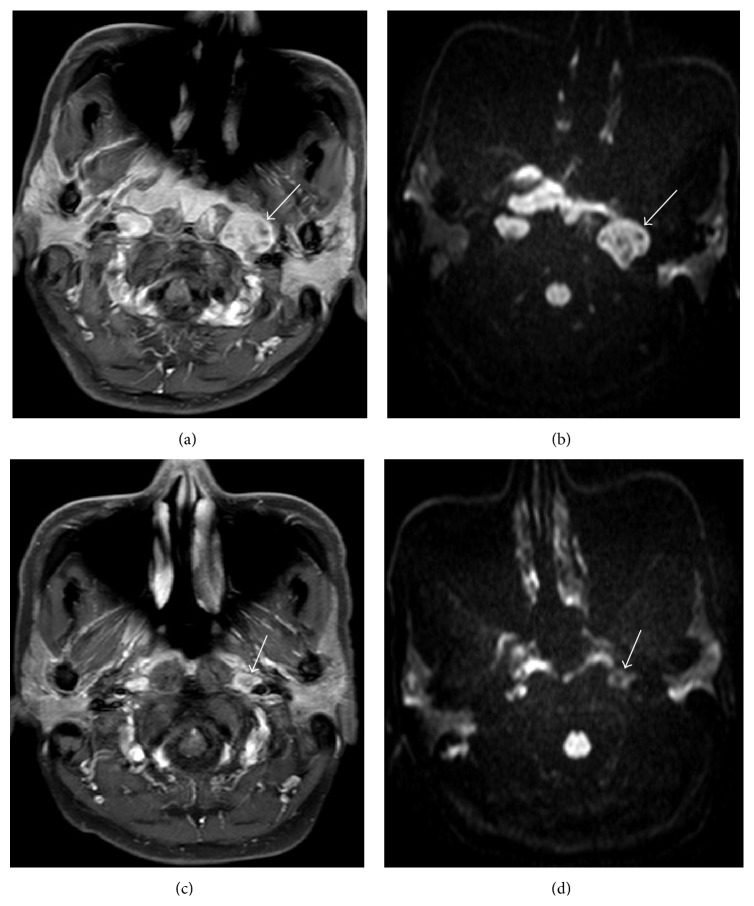
DW MRI findings in a 48-year-old female patient with NPC who responded to neoadjuvant chemotherapy. Pretreatment (a) axial contrast-enhanced T1-weighted image and (b) ADC maps showing an enlarged lymph node in the left retropharyngeal space (arrow). The mean pretreatment ADC of this lesion was 0.993 × 10^−3^ mm^2^/sec. Posttreatment (c) axial contrast-enhanced T1-weighted image and (d) ADC maps showing that the formerly enlarged right lymph node partially resolved; the volume of this lesion reduced by 83%. Neoadjuvant chemotherapy increased the mean ADC of this lesion to 1.383 × 10^−3^ mm^2^/sec.

Before neoadjuvant chemotherapy, the mean ADCs of responders were significantly lower than that of nonresponders (primary lesions: [0.798 ± 0.007] × 10^−3^ mm^2^/sec versus [1.019 ± 0.028] × 10^−3^ mm^2^/sec,* P* = 0.012; metastatic adenopathies: [0.964 ± 0.010] × 10^−3^ mm^2^/sec versus [1.135 ± 0.042] × 10^−3^ mm^2^/sec,* P* = 0.013). After completion of neoadjuvant chemotherapy, no significant difference in the mean ADCs was observed between responders and nonresponders (primary lesions: [1.274 ± 0.011] × 10^−3^ mm^2^/sec versus [1.366 ± 0.020] × 10^−3^ mm^2^/sec,* P* = 0.526; metastatic adenopathies: [1.354 ± 0.013] × 10^−3^ mm^2^/sec versus [1.427 ± 0.031] × 10^−3^ mm^2^/sec,* P* = 0.217). However, the mean percentage increases in the ADCs were significantly greater in responders than in nonresponders (primary lesions, 60.0% ± 2.4 versus 34.8% ± 3.2,* P* = 0.008; metastatic adenopathies, 40.7% ± 2.7 versus 26.5% ± 3.3,* P* < 0.001) (Figures 1 and 2) (Table 3). Additionally, the changes in the ADCs correlated with the change in tumor volume at follow-up MRI (primary lesions:* r* = 0.611,* P* < 0.001; metastatic adenopathies:* r* = 0.676,* P* < 0.001). Furthermore, a strong negative correlation was observed between the mean pretreatment tumor ADC and percentage change in tumor volume after chemotherapy (primary lesions:* r* = −0.570,* P* < 0.001; metastatic adenopathies:* r* = −0.423,* P* < 0.001).

### 3.4. ROC Curve Analysis

**Figure 3:**
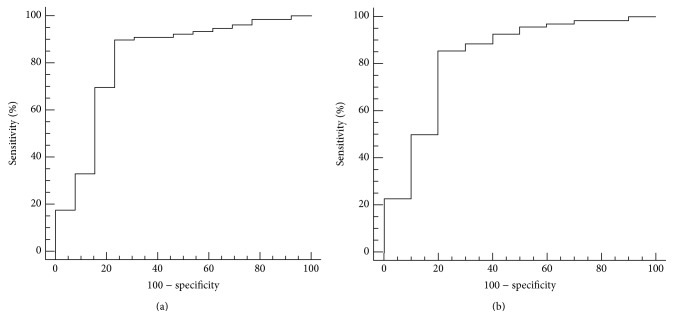
ROC curves of the ability of pretreatment primary tumor ADC (a) or metastatic adenopathy ADC (b) to predict the response to neoadjuvant chemotherapy in patients with nasopharyngeal carcinoma. The optimal pretreatment ADC cutoff values for the primary tumor and metastatic adenopathy were 0.897 × 10^−3^ mm^2^/sec and 1.031 × 10^−3^ mm^2^/sec, with areas under the ROC curves of 0.830 and 0.821, respectively.

The optimal pretreatment primary tumor ADC for differentiating responders from nonresponders using ROC curve analysis was 0.897 × 10^−3^ mm^2^/sec; this cutoff value had a sensitivity of 89.9% (71/79; 95% confidence interval: 81.0–95.5%), specificity of 76.9% (10/13; 95% confidence interval: 46.2–95.0%), and area under the empirical ROC curve of 0.821 (95% confidence interval: 0.727–0.893). The optimal pretreatment metastatic adenopathy ADC cutoff value for differentiating responders from nonresponders was 1.031 × 10^−3^ mm^2^/sec, which yielded a sensitivity of 85.7% (60/70; 95% confidence interval: 75.3–92.9%), specificity of 80.0% (8/10; 95% confidence interval: 44.4–97.5%), and area under the empirical ROC curve of 0.830 (95% confidence interval: 0.730–0.905) (Figure 3).

## 4. Discussion

This study demonstrated that, in patients with NPC, responders to neoadjuvant chemotherapy have significantly lower pretreatment ADCs than nonresponders, and a strong negative correlation exists between the mean pretreatment ADCs and percentage change in tumor volume on follow-up MRI. The patients with lower pretreatment ADCs had a better response to neoadjuvant chemotherapy compared to those with higher pretreatment ADCs, in accordance with other clinical studies [24–27]. In rectal cancer [24, 25] and breast cancer [26, 27], responders had lower ADCs before neoadjuvant chemotherapy than nonresponders. Therefore, we suggest that pretreatment ADCs could be used as a novel imaging biomarker to predict the response before neoadjuvant chemotherapy in patients with NPC. This could facilitate tailored therapeutic approaches in NPC, with some patients spared from ineffective and unnecessary treatment toxicities.

After completion of neoadjuvant chemotherapy, the percentage increases in the ADCs of responders were significantly greater than that of nonresponders, and the changes in the ADCs correlated with the change in tumor volume at follow-up MRI. Previous studies have shown that low pretreatment ADCs indicate viable tumor tissue with a high cellularity, whereas high ADCs reflect less metabolic tumor tissue with a low cellularity [28, 29]. Tumor tissue with a high rate of cellular proliferation is more sensitive to chemotherapy or radiotherapy, which acts by inducing cellular damage and lysis in proliferating cells followed by a reduction in cellular volume, thereby enhancing the diffusion of water molecules and increasing the ADCs values on DW MRI. However, tumors with a low cellularity are likely to be in a situation of hypoxia and ischemia, which reduces the delivery of chemotherapeutic agents to the tumor. Furthermore, cancer cells that have a slow rate of metabolism are less sensitive to cytotoxic chemotherapy or radiotherapy [28]. Therefore the changes in the ADCs of tumors with a low cellularity after completion of chemotherapy or radiotherapy will always be lower than the changes in the ADCs of tumors with a high cellularity. Interestingly, some studies have shown that large changes in ADCs during the early stages of treatment (after the first or second cycle of neoadjuvant chemotherapy or 1-2 weeks after the start of radiotherapy), which occur prior to changes in tumor diameter or volume, indicate a better response to treatment [12, 18, 30]. Therefore, in most malignant tumors, an obvious increase in the ADC is regarded as an important imaging biomarker of successful treatment [12–19, 24–27].

Over the last 10 years or so, DW MRI has been successfully employed in head and neck cancer to distinguish between residual disease or tumor recurrence and inflammation or necrosis after completion of (chemo)radiotherapy [31]. Additionally, pretreatment ADCs or changes in ADCs during (chemo)radiotherapy have been reported as useful markers for predicting locoregional failure or progression-free survival in head and neck carcinoma [17, 32]. Some early studies indicated the potential of ADCs for evaluating treatment response in head and neck carcinoma [12, 14, 18]. King et al. [14] reported that a large change in ADCs within two weeks of treatment was predictive of a better response to (chemo)radiotherapy. Kim et al. [18] observed that low pretreatment ADCs or a significant increase in ADCs within one week of treatment was indicative of a higher rate of locoregional remission after concurrent chemoradiation. Recently, a study of 31 patients with NPC showed that high ADCs and early large increases in ADCs after initiation of neoadjuvant chemotherapy were indicative of a better response to subsequent concurrent chemoradiotherapy [12]. However, to our knowledge, the use of pretreatment ADCs for predicting the response to neoadjuvant chemotherapy in patients with NPC or other head and neck carcinomas has not yet been reported. This study showed that patients with NPC and low pretreatment ADCs were more likely to respond to neoadjuvant chemotherapy, and large increases in ADCs after completion of neoadjuvant chemotherapy correlated with a better response to neoadjuvant chemotherapy. Therefore, assessment of ADCs may help identify patients who will fail to respond to neoadjuvant chemotherapy, thereby enabling individualized treatment planning and allowing some patients to avoid unnecessary chemotherapy and the associated toxicities.

It should be stressed that DW MRI was not performed after each cycle of neoadjuvant chemotherapy in this study. Other studies reported that changes in ADCs after the first cycle of neoadjuvant chemotherapy could provide more detailed information on tumor response [30]. Furthermore, the sample size in this study was relatively small, and most of the patients with NPC responded to neoadjuvant chemotherapy, which could result in statistical bias. Thus, one should be wary of applying the ADC cutoff values defined in this population for defining responders and nonresponders. In addition, pathologic confirmation of imaging findings is not possible in patients with NPC, who are typically treated with radiotherapy rather than surgery. Thus, determining the treatment response to chemotherapy based on anatomical MRI may be inaccurate. Therefore, we acknowledge that prospective, large cohort, and multicentre studies are necessary to confirm our findings and recommendations.

## 5. Conclusions

In NPC, patients with low pretreatment ADCs tended to respond better to neoadjuvant chemotherapy. Pretreatment ADCs have potential as a novel imaging marker to predict the response to neoadjuvant chemotherapy, which could facilitate individual therapeutic approaches and allow some patients with NPC to avoid ineffective chemotherapy and unnecessary treatment toxicities.

## Figures and Tables

**Table 1:** Response to neoadjuvant chemotherapy in 92 patients with NPC.

Response	Number of patients		
	NP (*n* = 92)	LN (*n* = 80)^∗^	Combination (NP + LN)
Responders			
Complete response	24 (26.1%)	44 (55.0%)	20 (21.7%)
Partial response	55 (59.8%)	26 (32.5%)	56 (60.9%)
Nonresponders			
Stable disease	13 (14.1%)	10 (12.5%)	16 (17.4%)
Progressive disease	0 (0%)	0 (0%)	0 (0%)

NP = nasopharynx; LN = regional neck lymph nodes.

^∗^12 patients with N0 disease were not included in the analysis.

**Table 2:** Clinicopathologic features of the responders and nonresponders.

Characteristic	Number of patients		*P*-value
	Responders (*n* = 76)	Nonresponders (*n* = 16)	
Sex			
Male	58	12	1.000
Female	18	4	
Age			
≥50 years	26	6	0.802
<50 years	50	10	
WHO pathologic type			
Type 1	1	0	0.834
Type 2	7	2	
Type 3	68	14	
Clinical stage (2010)			
III	43	9	0.997
IVA	24	5	
IVB	9	2	

WHO = World Health Organization.

**Table 3:** Tumor volume and ADCs of the primary tumor and metastatic adenopathies in 92 patients with NPC.

Characteristic	Number of patients		*P*-value
	Responders (*n* = 76)	Nonresponders (*n* = 16)	
Tumor volume (cm^3^)			
NP pretreatment	37.3 ± 2.7	36.2 ± 7.0	0.884
NP posttreatment	9.1 ± 1.2	23.7 ± 4.7	0.010
LN pretreatment	18.2 ± 1.7	20.0 ± 4.6	0.708
LN posttreatment	2.4 ± 0.5	14.3 ± 3.1	0.004
ADC (×10^−3^ mm^2^/sec)			
NP pretreatment	0.809 ± 0.009	0.953 ± 0.038	0.003
NP posttreatment	1.276 ± 0.007	1.306 ± 0.033	0.182
LN pretreatment	0.966 ± 0.010	1.121 ± 0.045	0.007
LN posttreatment	1.354 ± 0.009	1.355 ± 0.045	0.983
Increase in ADC (%)			
NP	58.5 ± 1.0	38.2 ± 3.1	<0.001
LN	40.4 ± 0.5	21.2 ± 1.3	<0.001

NP = nasopharynx; LN = regional neck lymph nodes.
